# Care of the Poor

**Published:** 1895-04

**Authors:** 


### SELECTIONS.

### Care of the Poor.

If the highest function of prevention is exercised, it must be done through channels that relate to the health of the poor. Disease can be prevented in a great measure by maintenance of nutrition, warmth and cleanliness. It is better to prevent sickness than to cure it. This aphorism holds good, viewed from the most severe standpoint of money economics. There is no surer way to breed disease than through the avenues of starvation and nakedness. Fuel, food and clothing are great sanitary allies, and should be dealt out with a liberal hand as preventive measures. This applies equally to public and private charity.—*Buffalo Med. and Surg. Journal.*

